# *Silene*, a versatile model system: from sex and genome evolution to ecology and speciation

**DOI:** 10.1111/nph.71153

**Published:** 2026-04-05

**Authors:** Sophie Karrenberg, Václav Bačovský, Andrea E. Berardi, Isabelle De Cauwer, Tatiana Giraud, Fanny E. Hartmann, Roman Hobza, Vojtěch Hudzieczek, Gabriel A.B. Marais, Jenna R. Miladin, Bengt Oxelman, Alexander S.T. Papadopulos, Daniel B. Sloan, Janet C. Steven, Helena Štorchová, Pascal Touzet, Fabienne Van Rossum

**Affiliations:** ^1^ Department of Ecology and Genetics Uppsala University Norbyvägen 18D 75236 Uppsala Sweden; ^2^ Department of Plant Developmental Genetics Institute of Biophysics of the Czech Academy of Sciences Kralovopolska 135 612 65 Brno Czech Republic; ^3^ Department of Biology James Madison University Harrisonburg VA 22807 USA; ^4^ Univ. Lille, CNRS, UMR 8198 – Evo‐Eco‐Paleo F‐59000 Lille France; ^5^ Ecologie, Société et Evolution, CNRS Université Paris‐Saclay, AgroParisTech F‐91190 Gif‐sur‐Yvette France; ^6^ LBBE—Laboratoire Biométrie et Biologie Evolutive CNRS/Université de Lyon 1 F‐69622 Villeurbanne France; ^7^ GreenUPorto—Sustainable Agrifood Production Research Centre, Departamento de Biologia, Faculdade de Ciências Universidade do Porto 4485‐661 Vairão Portugal; ^8^ Department of Biological Sciences University of Arkansas Fayetteville AR 72701 USA; ^9^ Department of Biology and Environmental Sciences, Gothenburg Global Biodiversity Centre University of Gothenburg Box 461 405 30 Göteborg Sweden; ^10^ Molecular Ecology and Evolution Group, School of Environmental and Natural Sciences Bangor University Bangor LL57 2UW UK; ^11^ Department of Biology Colorado State University Fort Collins CO 80523 USA; ^12^ Department of Biology, Chemistry and Environmental Science Christopher Newport University Newport News VA 23606 USA; ^13^ Institute of Experimental Botany Czech Academy of Sciences Rozvojová 263 Prague 16502 Czech Republic; ^14^ Meise Botanic Garden B‐1860 Meise Belgium; ^15^ Service général de l'Enseignement supérieur et de la Recherche scientifique Fédération Wallonie‐Bruxelles B‐1080 Brussels Belgium

**Keywords:** biotic interactions, breeding systems, cyto‐nuclear interactions, *Microbotryum*, repeated adaptive evolution, sex chromosomes, speciation continuum

## Abstract

Fundamental and applied research in evolutionary biology benefits from the use of model systems in which approaches from disparate disciplines can be integrated. Here, we review recent progress in evolutionary research on the long‐standing model system *Silene*, a large genus with a well‐resolved phylogeny and newly available, expanded genomic resources. We report how studies using *Silene* have pioneered advances in the understanding of the structure and function of sex chromosomes and the rapid evolution of plant organelles. *Silene* was instrumental for elucidating the causes and consequences of shifts in sexual systems, in particular between gynodioecy and dioecy. Investigations of *Silene* species and associated anther‐smut fungi have further led to major insights into host specialization and coevolution in plant–pathogen systems. Moreover, *Silene* has recently developed into a promising model system for the understanding of evolutionary responses to abiotic conditions, of pollinator‐mediated evolution of flower advertising traits and of the drivers of speciation. We outline open questions for which the *Silene* system is particularly suitable, including the use of previously underexplored comparative approaches.

## Résumé

La recherche fondamentale et appliquée en biologie évolutive bénéficie de l’utilisation de modèles d’étude qui intègrent des approches issues de disciplines diverses. Nous présentons ici une synthèse des progrès récents en recherche évolutive sur un modèle étudié de longue date, le genre *Silene*, un genre important dont la phylogénie est bien résolue et dont les ressources génomiques ont récemment été enrichies. Nous montrons comment les études menées sur *Silene* ont permis des avancées majeures dans la compréhension de la structure et de la fonction des chromosomes sexuels et de l’évolution rapide des organites chez les plantes. Le modèle *Silene* a joué un rôle déterminant dans l’élucidation des causes et des conséquences des changements de systèmes sexuels, notamment entre la gynodioécie et la dioécie. Les recherches sur les espèces de *Silene* et leurs champignons associés (*Microbotryum*, responsables de la maladie du charbon des anthères) ont également permis des apports essentiels dans notre compréhension de la spécialisation à l’hôte et la coévolution dans les systèmes plantes‐pathogènes. De plus, le genre *Silene* est récemment devenu un modèle prometteur pour comprendre les réponses évolutives aux conditions abiotiques, l’évolution des caractères floraux induite par les pollinisateurs et les mécanismes de la spéciation. Nous présentons des questions ouvertes pour lesquelles le système *Silene* est particulièrement adapté, notamment pour l’utilisation d’approches comparatives.

**Table:** 

	Contents	
	Summary	3614
I.	Introduction	3614
II.	*Silene*, a large genus with a wide diversity	3614
III.	Structural and functional genomics: a breakthrough in sex chromosome studies	3615
IV.	Rapid divergence of organellar genomes and coevolutionary interactions with the nucleus	3619
V.	The castrating anther‐smut fungi: plant–pathogen host specialization and coevolution	3620
VI.	Evolution of sexual systems: from combined to separate sexes	3621
VII.	Ecological and evolutionary responses to abiotic environments	3622
VIII.	Pollinator‐mediated evolution: benefits and costs	3623
IX.	The speciation continuum in *Silene*: understanding how species form	3624
X.	Conclusions and perspectives	3625
	Acknowledgements	3625
	References	3626

## I. Introduction

**Fig. 1:**
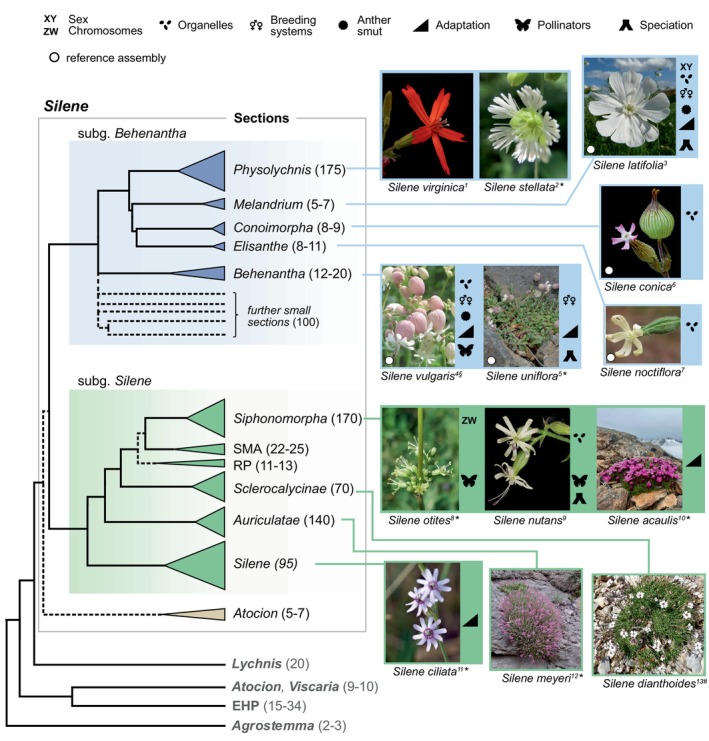
Phylogenetic relationships among genera within the tribe *Sileneae* and of sections within *Silene* after Jafari *et al*. (2020), with photographs of example species. Generic names are given in bold type and section names in regular type; numbers in parentheses denote the approximate number of currently recognized taxonomic species. Dashed branches indicate inconclusive branchings and the width of the triangles approximates the relative diversity within taxa. Species used for recent advances in the seven research areas and treated in this review are marked with symbols. White circles indicate that a whole‐genome reference assembly is available as of August 2025. SMA, *Silene* sections *Sclerophyllae*, *Muscipula*, and *Arenosae*; RP, *Silene* sections *Rigidulae* and *Portenses*; EHP, *Silene* sections *Eudianthe*, *Heliosperma* and *Petrocoptis*. Photographs by Andrea Berardi (^1^), Rafael Urbina Casanova (^2^), Michaël Falkowski (^3^), D. Gordon E. Robertson (^4^), Chris Close (^5^), Daniel J. Parmentier (^6^), Daniel B. Sloan (^7^), Julia Wanz (^8^), Maarten Strack van Schijndel (^9^), C.S.Yampae (^10^), Riccardo Molajoli (^11^), Alexander Ivanov (^12^), Ekaterina Kropocheva (^13^) displayed as cropped versions under CC BY‐NC (*), CC BY 4.0 (^§^) and CC BY‐SA 3.0 (^#^) licenses.

The study of evolutionary processes requires an integration across biological disciplines, spanning genomics, bioinformatics, cytogenetics and field ecology, and model systems. Here, we review how recent studies on the large plant genus *Silene*, a long‐standing model system, have contributed to recent advances in evolution and ecology, with a focus on work conducted since the last broad review of research work in *Silene* (Bernasconi *et al*., 2009). Methodological advances in sequencing technology and in structural and functional genomics have greatly expanded the scope of research conducted in this system. These developments, as well as long‐standing organismal and ecological expertise, have allowed for *Silene* to become a leading system for the study of sex chromosomes (Yue *et al*., 2023; Akagi *et al*., 2025; Moraga *et al*., 2025), organelles (Sloan *et al*., 2012a, 2014a), breeding system evolution (Štorchová *et al*., 2018; Muyle *et al*., 2021b) and host specialization and coevolution in plant–pathogen systems (Hartmann *et al*., 2019). In addition, *Silene* research is starting to make very promising contributions to the understanding of responses to abiotic conditions (Doak & Morris, 2010; Papadopulos *et al*., 2021; Gramlich *et al*., 2022), plant–pollinator interactions (Prieto‐Benítez *et al*., 2017; Berardi *et al*., 2022) and speciation (X. Liu *et al*., 2020; Postel *et al*., 2025). Here, we review recent discoveries in these seven areas (Fig. 1). However, this article cannot be exhaustive due to space constraints and we apologize to colleagues whose work and research areas we were unable to cover. We further outline open questions for which the *Silene* system is a particularly suitable biological model, including the use of previously underexplored comparative approaches in this large genus.

## II. *Silene*, a large genus with a wide diversity

*Silene* (Linnaeus, 1753) is a highly diverse genus consisting of currently *c*. 850 taxonomic species with native distributions on all continents except Australia and Antarctica (Jafari *et al*., 2020 and references therein). Additional species are still being described, especially from Central and Southwest Asia (IPNI, 2025). Most *Silene* species are diploid, but many polyploids are found in the section *Physolychnis* (Quatela *et al*., 2025). Known haploid genome sizes range from 0.9 to 3.3 Gbp (Pellicer & Leitch, 2020). The taxonomy of *Silene* and its close relatives has a long and controversial history due to the high level of morphological similarity within and across subgenera, suggesting many potential cases of repeated evolution (Jafari *et al*., 2020; Miladin *et al*., 2022; Quatela *et al*., 2025). The revised taxonomy of Jafari *et al*. (2020) recognizes phylogenetically well‐supported infrageneric taxa including two well‐supported large clades, the subgenera *Behenantha* and *Silene* (Fig. 1). The former subgenus is characterized by a hypothetical ancient and rapid radiation, which makes its basal dichotomous resolution difficult to resolve (Jafari *et al*., 2020). The dashed branches in the subgenus *Behenantha* in Fig. 1 indicate *c*. 20 relatively species‐poor groups involved in this radiation. By contrast, the subgenus *Silene* is better resolved basally and includes sections, which diversified more recently (Fig. 1). *Lychnis* is closely related to and sometimes included in *Silene* because of ambiguous resolution in studies with smaller data sets, but recent results based on transcriptomics (Cangren *et al*., 2024; Cangren & Oxelman, unpublished data) strongly suggest *Lychnis* to be the sister group to *Silene*.

**Fig. 2:**
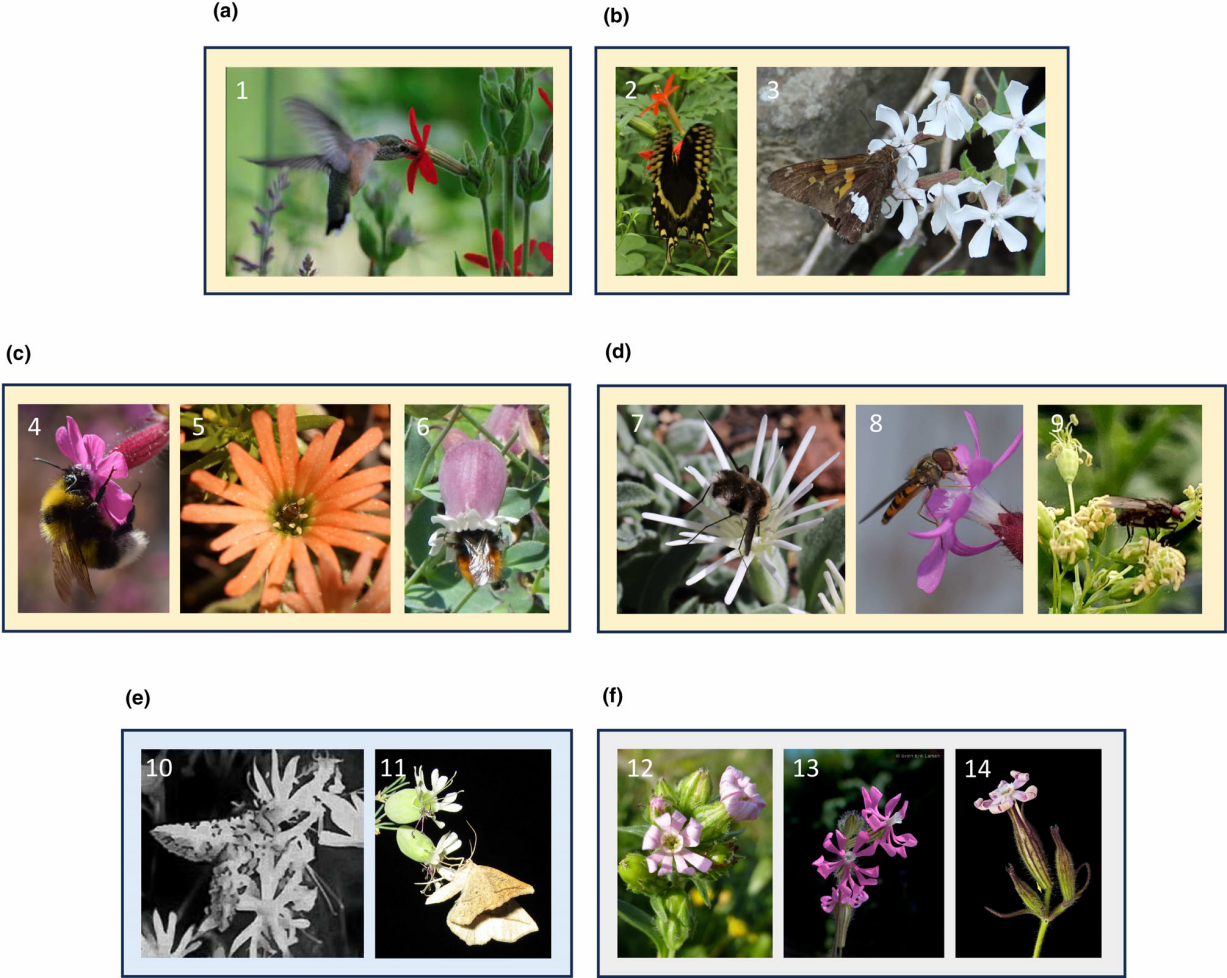
Primary pollinator functional groups in *Silene*: diurnal pollination (yellow frames), by (a) hummingbirds (Ruby‐throated hummingbird on *Silene regia*); (b) butterflies (*Papilio* sp. on *Silene subciliata* and *Epargyreus clarus* on *Silene caroliniana*); (c) bees (*Bombus* sp. on *Silene dioica*, solitary bee on *Silene salmonacea* and on *Silene vulgaris*); (d) flies (*Bombylius* sp. on *Silene nelsonii*, Syrphidae on *Silene dioica* and Muscidae on *Silene otites*); (e) nocturnal pollination by moths (blue frame; *Hadena albimacula* on *Silene nutans* and *Aspitates gilvaria* on *Silene vulgaris*); and (f) selfing species (gray frame; *Silene apetala*, *Silene colorata* and *Silene noctiflora*). Photographs by Darin Ziegler (1), Andrea Berardi (2, 3, 5, 6), Paul Moreau (4, 8), Jenna Miladin (7), Fabienne Van Rossum (9), Camille Cornet (10), James D. Stone (11), Sami Tamson (12), Manuel A.M.‐Madrid (13), and Maarten Strack van Schijndel (14).

*Silene* species are small‐ to mid‐sized forbs and have a generally similar morphology: Leaves are opposite and the white, pink, bluish or red flowers with five petals are arranged in cymose inflorescences (Jafari *et al*., 2020; Fig. 1). Many species have basal leaf‐rosettes, and some have inflated calyces, for example the bladder campion, *Silene vulgaris* (Fig. 1). *Silene* includes annuals, biennials and perennials. Some of the latter are long‐lived, such as the arctic‐alpine cushion plant *Silene acaulis* (Fig. 1). Habitats range from dry to mesic open grassland and forest habitats, to extreme environments, for example coastal cliffs, high‐altitude screes and heavy‐metal rich soils, usually with low levels of competition from other plants (Fig. 1; Papadopulos *et al*., 2021). While many *Silene* species are hermaphroditic, *Silene* is well‐known for gynomonoecy, gynodioecy and dioecy (Casimiro‐Soriguer *et al*., 2015), for its diversity in plant–pollinator interactions (Fig. 2), and for plant venereal diseases caused by fungi (Hartmann *et al*., 2019). Among the dioecious species, both XY and ZW sex chromosomes have been described (Fig. 1; will be discussed later).

## III. Structural and functional genomics: a breakthrough in sex chromosome studies

Dioecy and sex chromosomes have independently evolved at least three times within *Silene*, in the sections *Melandrium* and *Otites* (*Siphonomorpha* s.l.) and in *S. acaulis* (Bernasconi *et al*., 2009). Distinct sex chromosome architectures have formed in *Melandrium* and *Otites*; the sex determination type is unknown in *S. acaulis*. While *Silene latifolia* sex chromosomes are heteromorphic, with the Y being larger than the X (Akagi *et al*., 2025; Moraga *et al*., 2025, and references therein) sex chromosomes in *Otites* are homomorphic with much smaller nonrecombining regions than in *S. latifolia* (Balounova *et al*., 2019; Martin *et al*., 2019). In *Otites*, the ZW sex determination system is the ancestral one, and transitions to XY systems in some species (*Silene colpophylla* and *Silene pseudotites*) indicate sex‐chromosome turnover – an evolutionary process in which sex‐determining genes are replaced over time within a lineage (Balounova *et al*., 2019; Martin *et al*., 2019).

The giant size of the *S. latifolia* Y chromosome was initially seen as an advantage by the research community, since it enabled the first discovery of sex chromosomes in vascular plants and simplified their study through cytological and cytogenetic methods (Blackburn, 1923). However, assembling the giant, repeat‐rich Y chromosome later proved challenging. Therefore, the *Silene* research community developed innovative, nonstandard approaches to identify sex‐linked genes (Box 1; Hobza *et al*., 2024). Applied to *Silene* species and other dioecious plants, these methods led to major breakthroughs – the discovery of the plant sex‐determining genes, and the demonstration of dosage compensation in plants. The advent of long‐read sequencing is now transforming the field, making it possible to assemble large and complex Y chromosomes, as exemplified by the recent high‐quality assembly of a male *S. latifolia* genome (Akagi *et al*., 2025; Moraga *et al*., 2025).

Box 1 **Recent methodological advances and available genomic resources in *Silene***

**Fig. B1:**
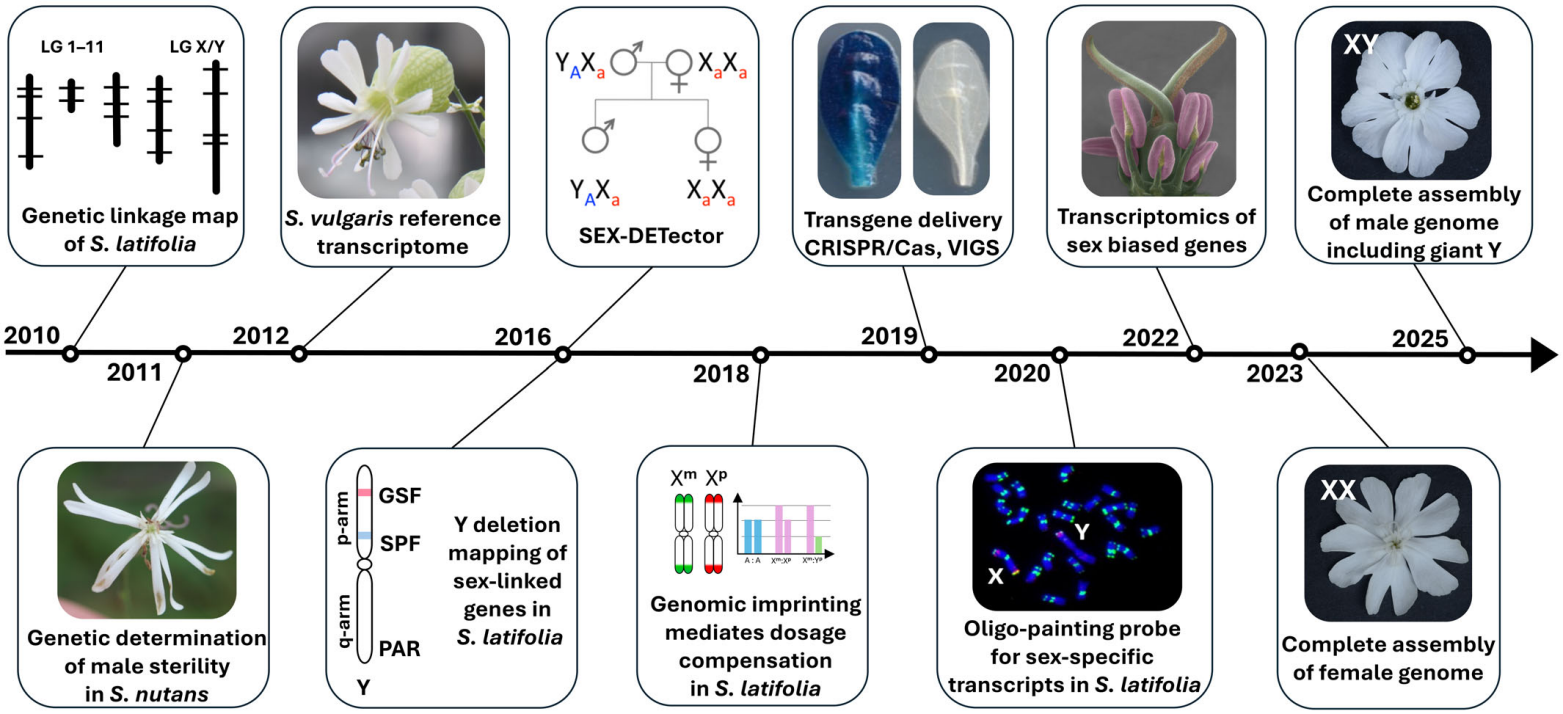
Schematic representation of recent methodological advances and the availability of genomic resources in *Silene*, particularly in *Silene latifolia*, *Silene vulgaris* and *Silene nutans*. Photos and drawings: Helena Štorchová, Václav Bačovský, Roman Hobza, Vojtěch Hudzieczek and Gabriel Marais; *Silene nutans* photograph by Bernd Haynold, via Wikimedia Commons (CC BY 2.5).

Over the years, significant genomic resources have been developed (Fig. B1; Table 1). These include a genetic linkage map in *Silene latifolia* (Delph *et al*., 2010), a transcriptome assembly in *Silene vulgaris* (Sloan *et al*., 2012c), a detailed genetic map of the Y chromosome (Kazama *et al*., 2016), and the recent complete assembly of both male and female genomes of *S. latifolia* (Yue *et al*., 2023; Akagi *et al*., 2025; Moraga *et al*., 2025). Key breakthroughs for functional studies such as *Agrobacterium*‐mediated transformation and virus‐induced gene silencing enabled the community to investigate regulatory pathways of flower development in *S. latifolia* (Fujita *et al*., 2019; Hudzieczek *et al*., 2019). Additionally, studies on dosage compensation mechanisms have revealed genomic imprinting mechanisms as a potential mediator, helping to balance gene expression between sexes (Muyle *et al*., 2018). The development of the SEX‐DETector approach has further enabled precise studies of sex‐linked genes, providing insights into the evolutionary dynamics of sex chromosomes (Muyle *et al*., 2016). Chemical genetics, a method that uses small molecules to study gene function or gene product, has emerged as a valuable tool for modulating flower development, allowing for targeted manipulation of developmental pathways in *S. latifolia* (Bačovský *et al*., 2022). Moreover, the design of oligo‐painting probes has become a promising technique for cytogenetic mapping, improving the visualization of chromosomal rearrangements in *Silene* species and confirming the position of Y‐linked scaffolds (reviewed in Hobza *et al*., 2024).

### 1. Identification of sex‐linked genes through the development of new analytic tools

*Silene* was the subject of the first studies using transcriptomics to identify hundreds of plant sex‐linked genes (Bergero & Charlesworth, 2011; Chibalina & Filatov, 2011; Muyle *et al*., 2012). These methodologies paved the way for analyzing sex chromosomes without relying on complete genome assemblies (Box 1; Hobza *et al*., 2024). The development of methods leveraging data from parents and separately pooled male and female offspring enabled the analysis of complete transcriptome profiles in controlled crosses (LINKXY; Michalovova *et al*., 2015). A major advance followed with the introduction of SEX‐DETector, and its companions SEX‐DETector++ and SDpop, which are model‐based tools that analyze allele transmission patterns or deviations from Hardy–Weinberg expectations to distinguish sex‐linked from autosomal genes while accounting for genotyping errors (Muyle *et al*., 2016; Käfer *et al*., 2021). Unlike other methods that rely on using arbitrary cut‐offs to remove wrongly inferred sex‐linked Single Nucleotide Polymorphisms (SNPs) corresponding to genotyping errors, these tools return posterior probabilities, offering a more robust and probabilistic identification of sex‐linked genes. This has significantly advanced the field by providing efficient and reliable ways to identify and analyze sex chromosomes (Marais *et al*., 2025).

### 2. The sequencing of the giant Y chromosome in *Silene latifolia*

With the rapid advancement of long‐read sequencing technologies, recent efforts have led to the completion of several *S. latifolia* genome assemblies, which have enabled the resolution of structural rearrangements between the X and Y chromosomes, the delimitation of evolutionary strata formed through at least four successive events of recombination suppression and the reconstruction of their chronological order (Yue *et al*., 2023; Akagi *et al*., 2025; Moraga *et al*., 2025). The evolution of *S. latifolia* sex chromosomes started with the differentiation of a small region carrying the gynoecium‐suppression factor sex‐determining gene (*c*. 11 million years ago (Ma)), and the first stratum then likely arose through two inversions, on the X and the Y chromosomes, roughly at the same time, *c*. 11 Ma (Yue *et al*., 2023; Akagi *et al*., 2025; Moraga *et al*., 2025). The second stratum was formed later, accompanied by a global expansion of the pericentromeric regions due to a massive accumulation of transposable elements (TEs), in both the sex chromosomes and the wider *S. latifolia* genome *c*. 5 Ma (Yue *et al*., 2023; Akagi *et al*., 2025; Moraga *et al*., 2025). The third stratum corresponds to a DNA fragment that was initially within the first stratum but was lost from the Y and later regained by a recent duplicative translocation from the X, resetting the X‐Y divergence to zero *c*. 4.4 Ma and restoring X‐Y synteny there (Moraga *et al*., 2025). A small fourth stratum, close to the pseudoautosomal region, has specifically evolved in *S. latifolia* and is not present in its closest relative, *Silene dioica* (Yue *et al*., 2023; Filatov, 2024). Interestingly, the *Microbotryum* fungi causing anther‐smut disease in *Silene* plants also possess mating‐type chromosomes that resemble sex chromosomes with evolutionary strata (Box 2).

Box 2 ***Microbotryum* fungi: biology, host specialization and mating‐type chromosomes**

**Fig. B2:**
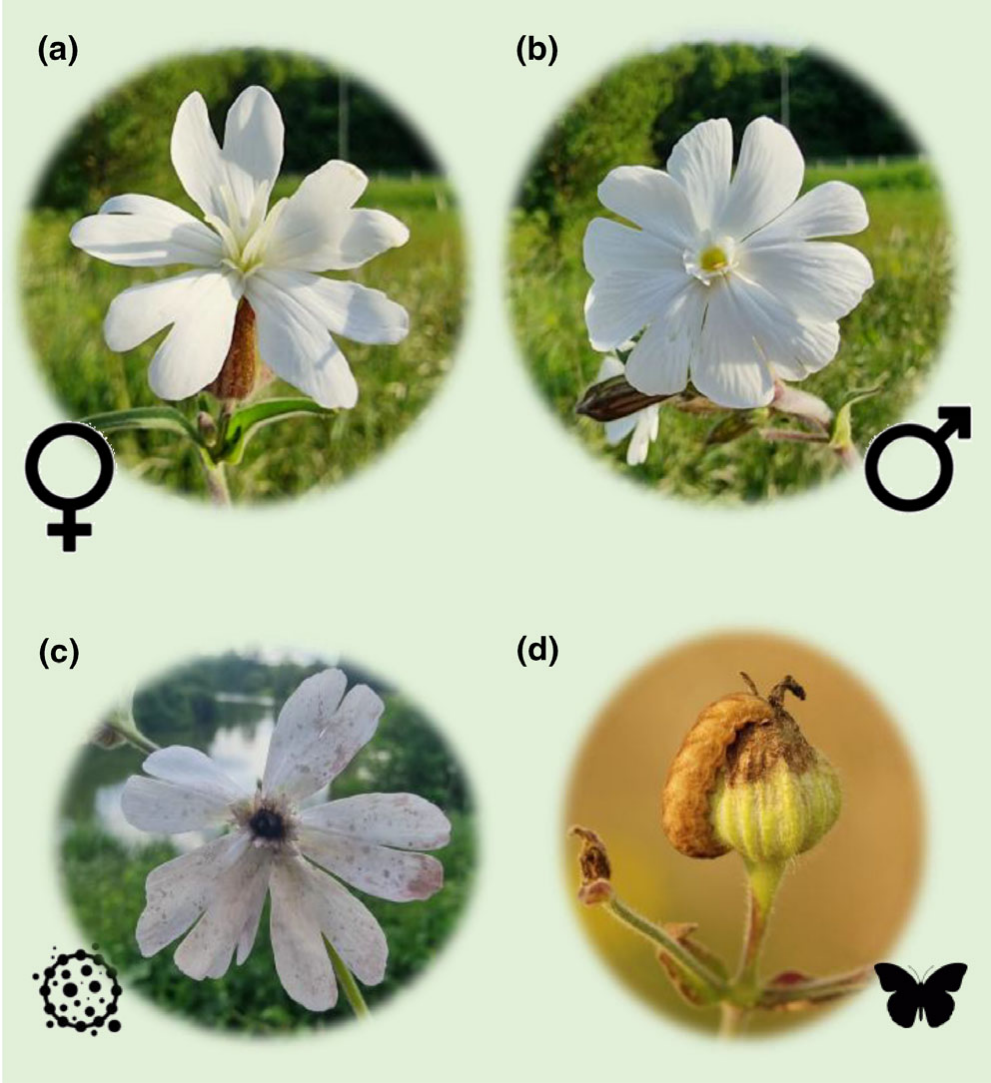
*Silene latifolia* and its enemies: (a) female, (b) male and (c) diseased flowers (castrated by the anther‐smut *Microbotryum* fungus), and (d) caterpillar of the seed‐eater *Hadena bicruris* on a capsule (photographs by Thierry Darras and Margriet Louwen).

Hundreds of *Microbotryum* species, causing the anther‐smut disease, are pathogens of perennial *Silene* species, castrating their hosts through manipulation of the hosts' reproductive organs; these fungi produce their spores in flower anthers and abort ovaries (Fig. B2; Hood *et al*., 2010). In dioecious plant species, the fungi transform females into male‐like plants bearing anthers full of spores, which has intrigued Charles Darwin and can inform on the genetics of plant sex determinism (Fujita *et al*., 2023). When studied with genetic markers, most *Microbotryum* species were found to be specialized on one host species of the Caryophyllaceae (Hartmann *et al*., 2019). Reciprocally, most host species have a single *Microbotryum* pathogen species, with the notable exception of *Silene vulgaris* on which three different anther‐smut species coexist (Abbate *et al*., 2018). Inversions on autosomes have been suggested to facilitate their speciation and host specialization (Hartmann *et al*., 2020). *Microbotryum* fungi have become a major model for studying speciation in plant pathogenic fungi, revealing gradual evolution of post‐mating isolation (Hartmann *et al*., 2019), divergence with continuous and ongoing gene flow in some species pairs (Hartmann *et al*., 2020), and secondary contact after geographic separation in other species pairs (Gladieux *et al*., 2011).

*Microbotryum* species have been the first fungi in which dimorphic mating‐type chromosomes have been described (Hood *et al*., 2004). In addition, evolutionary strata have repeatedly evolved across the genus (Lucotte *et al*., 2025), despite the lack of differentiated sexes, providing evidence that sexual antagonism is not the sole selection force able to generate progressive recombination suppression (Lucotte *et al*., 2025). *Microbotryum* mating‐type chromosomes can be highly degenerated, with transposable element accumulation (Carpentier *et al*., 2022; Duhamel *et al*., 2023) and recessive deleterious alleles, lethal at the haploid stage (Hood & Antonovics, 2000).

*Silene latifolia* has historically played a key role in supporting the two‐gene model, postulating that dioecy and sex chromosomes evolve through recombination suppression genetically linking together a male‐fertility and a female‐sterility mutation on the Y (Charlesworth & Charlesworth, 1978). In line with this hypothesis, the oldest stratum in *S. latifolia* harbors *WUSCHEL1* (*WUS1*) and *CLAVATA3* (*CLV3*), two instrumental genes for flower development in male and female, respectively (Moraga *et al*., 2025). *WUS1* has a carpel‐promoting role and *CLV3* is stamen‐promoting. A functional *CLV3* copy has only remained on the Y chromosome while a functional *WUS1* copy is only present on the X. A higher dosage of *CLV3* than that of *WUS1* could explain male development in XY individuals, while the opposite situation would result in female development in XX individuals (Kazama *et al*., 2022; Akagi *et al*., 2025; Moraga *et al*., 2025).

### 3. Y chromosome decay in *Silene latifolia*

The degeneration (decay) of the Y chromosome is a key aspect of sex chromosome evolution, reflecting the loss of gene function and expression due to the accumulation of deleterious mutations or TE insertions. X‐linked genes without an expressed Y counterpart (X‐hemizygous genes) were identified using their specific segregation patterns, providing the first estimates of Y gene decay in *S. latifolia* (Bergero & Charlesworth, 2011; Bergero *et al*., 2015; Muyle *et al*., 2016). The proportion of X‐hemizygous genes was later estimated to be 40% (Papadopulos *et al*., 2015), while the full sequencing of the Y revealed that this number was as high as 50–60% (Akagi *et al*., 2025; Moraga *et al*., 2025). In *S. latifolia*, Y‐linked alleles tend to have lower expression than their X counterparts (Muyle *et al*., 2012), which also constitutes degeneration. Y chromosome degeneration, in terms of both gene loss and dysregulation, is mostly caused by the accumulation of TEs (Puterova *et al*., 2018; Muyle *et al*., 2021a).

### 4. X chromosome dosage compensation

X chromosome dosage compensation is a regulatory mechanism that restores optimal expression despite the loss of genes or their expression in males following Y degeneration. While dosage compensation had long been recognized in animals, with many RNA‐seq studies suggesting that it is pervasive, it was only convincingly demonstrated in plants recently, in *S. latifolia* (Muyle *et al*., 2012, 2018, 2022). Dosage compensation appears to have evolved through an incomplete form in *S. latifolia*, overexpressing the maternal X chromosome in both sexes via genomic imprinting, which may be suboptimal (Muyle *et al*., 2012, 2018; Papadopulos *et al*., 2015; but see Krasovec *et al*., 2019). Interestingly, the compensated genes tend to be located in the oldest, most degenerated stratum of the sex chromosomes (Akagi *et al*., 2025), with few or perhaps no compensated genes in the youngest strata. This is not consistent with the regulatory model for the evolution of sex chromosomes, which proposes that very early dosage compensation is required for the evolution of recombination suppression on sex chromosomes (Lenormand & Roze, 2022). The next step is to explore the epigenetic and molecular mechanisms underlying dosage compensation and its evolution using closely related gynodioecious species within section *Behenantha* as an outgroup.

## IV. Rapid divergence of organellar genomes and coevolutionary interactions with the nucleus

The known roles of mitochondrial genetics in cytoplasmic male sterility (CMS) and breeding system diversity (will be discussed later) sparked early interest in the organelle genomes of *Silene* and in their roles in cell metabolism, organismal biology, and evolution. Sequencing of mitochondrial and plastid genomes (mitogenomes and plastomes, respectively) in *Silene* over the past 15 years (Table 1) has identified numerous distinctive features. This has given insights into organellar modes of inheritance and rapid evolution of sequence, structure and gene content, making *Silene* a key model for studying organelle genome evolution and cytonuclear interactions.

**Table 1:** *Silene* species with sequenced nuclear (A), mitochondrial (B) and plastid (C) genomes.

A. Nuclear genome assemblies	
Species	References
*Silene conica*	Fields *et al*. (2023)
*Silene dioica*	Akagi *et al*. (2025)
*Silene latifolia*	Yue *et al*. (2023); Akagi *et al*. (2025); Moraga *et al*. (2025)
*Silene noctiflora*	Williams *et al*. (2021)
*Silene uniflora*	Osborne *et al*. (2026)
*Silene vulgaris*	Akagi *et al*. (2025); Moraga *et al*. (2025)

B. Mitochondrial genomes		
Species	Accession	References
*Silene conica*	JF750490–JF750629	Sloan *et al*. (2012a)
*Silene latifolia*	HM562727	Sloan *et al*. (2010)
*Silene noctiflora*	JF750431–JF750489	Sloan *et al*. (2012a)
*Silene vulgaris*	JF750427–JF750430	Sloan *et al*. (2012a)

C. Plastid genomes		
Species	Accession	References
*Silene adscendens*	MN365968	del Valle *et al*. (2019)
*Silene alexandrae*	ON598354	Song *et al*. (2023)
*Silene aprica*	MK397897	Yao *et al*. (2019)
*Silene asclepiadea*	MN078083	Unpublished
*Silene atrocastanea*	MN097702	Tang *et al*. (2019)
*Silene atsaensis*	ON527036	Liu *et al*. (2024)
*Silene caespitella*	ON527037	Liu *et al*. (2024)
*Silene cambessedesii*	MN365973	del Valle *et al*. (2019)
*Silene capitata*	KT727930	Kang *et al*. (2017)
*Silene chalcedonica*	KF527886	Sloan *et al*. (2014a)
*Silene chodatii*	MN097705	Tang *et al*. (2019)
*Silene conica*	JF715054	Sloan *et al*. (2012b)
*Silene conoidea*	KF527885	Sloan *et al*. (2014a)
*Silene delavayi*	MN097707	Tang *et al*. (2019)
*Silene firma*	OP353920	Unpublished
*Silene gonosperma*	ON598349	Song *et al*. (2023)
*Silene gracilicaulis*	MN097710	Tang *et al*. (2019)
*Silene graminifolia*	ON598348	Song *et al*. (2023)
*Silene jenisseensis*	MN723869	Ling (2020)
*Silene kiusiana*	MH490802	Yoon *et al*. (2019)
*Silene latifolia*	JF715055	Sloan *et al*. (2012b)
*Silene lhassana*	ON527038	Liu *et al*. (2024)
*Silene lineariloba*	MN097712	Tang *et al*. (2019)
*Silene lithophila*	ON598346	Song *et al*. (2023)
*Silene littorea*	MN365975	del Valle *et al*. (2019)
*Silene melanantha*	MN097714	Tang *et al*. (2019)
*Silene nana*	ON598344	Song *et al*. (2023)
*Silene noctiflora*	JF715056	Sloan *et al*. (2012b)
*Silene olgiana*	ON598342	Song *et al*. (2023)
*Silene paradoxa*	KF527887	Sloan *et al*. (2014a)
*Silene psammitis*	MN365988	del Valle *et al*. (2019)
*Silene repens*	ON598340	Song *et al*. (2023)
*Silene stewartiana*	MN097717	Tang *et al*. (2019)
*Silene stockenii*	MN365992	del Valle *et al*. (2019)
*Silene tatarinowii*	MN097720	Tang *et al*. (2019)
*Silene uniflora*	KY562597	Dann *et al*. (2017)
*Silene viscidula*	MN097721	Tang *et al*. (2019)
*Silene vulgaris*	JF715057	Sloan *et al*. (2012b)
*Silene wilfordii*	KT727929	Kang *et al*. (2017)

### 1. Dynamic evolution of organelle genome size and structure

*Silene* mitogenomes vary more than 40‐fold in size (from 253 kb to over 11 Mb), meaning that >90% of the known range of mitogenome sizes in all eukaryotes is captured within this one genus (Sloan *et al*., 2012a). Although it has long been known that plant mitogenomes are large and variable in size (Ward *et al*., 1981), discoveries in *Silene* greatly expanded this range. The structural variation among *Silene* mitogenomes is equally remarkable. While some can be represented as a single ‘master circle’ akin to that found in most angiosperms, others have been fragmented into dozens of different chromosomes (Sloan *et al*., 2012a), with extensive variation in the set of chromosomes even among individuals within a species (Wu & Sloan, 2019). *Silene conica* and *Silene noctiflora* were the first plants to have highly multichromosomal mitogenomes discovered, but this structural organization is now known to be a recurring theme across angiosperm diversity (Wu *et al*., 2022). *Silene* plastomes do not exhibit the same degree of size variation, but they do show structural instability in a similar subset of species with extreme mitogenome expansion and fragmentation (Sloan *et al*., 2014a), posing an open question about the evolutionary pressures and molecular mechanisms that link the fate of these two organelle genomes in *Silene* and beyond.

### 2. Extreme variation in rates of mitochondrial and plastid sequence evolution

Comparisons of mitochondrial gene sequences led to the unexpected finding that *Silene* species can differ by orders of magnitude in rates of nucleotide substitution. This variation is thought to be driven by changes in underlying mitochondrial mutation rates because effects are the strongest at synonymous sites, which should be relatively free from selection (Mower *et al*., 2007; Sloan *et al*., 2009), and because high‐fidelity sequencing has detected large differences among species in frequencies of *de novo* mutations in tissue samples (Broz *et al*., 2021). Accelerated mitochondrial evolutionary rates have been observed in numerous plant lineages (Mower *et al*., 2007), but the major differences in mitochondrial evolutionary rates even among closely related *Silene* make the genus an especially valuable study system. Even in *Silene* species with rates of mitochondrial sequence evolution more typical of plant mitogenomes, there are some gene‐specific accelerations (Sloan *et al*., 2009) – a phenomenon that has since been found to occur to an even greater degree in other angiosperm lineages (F. Liu *et al*., 2020).

The accelerated mitochondrial mutation rates in *S. conica* were found to be associated with an exceptionally low mitochondrial genome copy number (Broz *et al*., 2021). This observation was recently extended across seed plants, leading to the discovery of a general negative relationship between mitochondrial mutation rate and genome copy number and suggesting that the availability of additional templates for recombinational repair might be a key determinant of plant mitochondrial mutation rates (Zwonitzer *et al*., 2024).

Rates of plastome sequence evolution also vary within *Silene*, with drastic accelerations often occurring in the same species that show atypical mitogenome evolution (Sloan *et al*., 2014a). However, the specific patterns of plastome rate variation are very different from those in the mitogenome and show evidence for large changes in the selection regime (rather than any obvious differences in mutation rate). Changes in selection appear to affect only a subset of plastid genes that do not encode components of photosynthetic enzyme complexes and instead function in processes such as fatty acid biosynthesis, protein turnover, transcription, translation and protein import (Erixon & Oxelman, 2008; Sloan *et al*., 2014a; Broz *et al*., 2021). Strikingly, substitution rates have accelerated independently in the same set of genes in multiple *Silene* species (including *clpP1*, *ycf1*, *ycf2*, and genes encoding ribosomal and RNA polymerase subunits), resulting in a recurring but still largely unexplained pattern that is found in many other angiosperm lineages (Forsythe *et al*., 2021).

Heterogeneous rates of sequence evolution in organelle genomes have made *Silene* a model for addressing general evolutionary questions. Substitution rate accelerations in *Silene* are correlated with increased mitogenome size but also with loss of features such as RNA editing sites, introns and entire genes (Sloan *et al*., 2012a), providing tests of major hypotheses that seek to explain the evolution of genome size and architecture (Brandvain & Wade, 2009; Smith, 2016). The natural evolutionary rate variation in *Silene* has also provided opportunities for testing long‐standing hypotheses that changes in organelle genomes can create selection for coevolutionary responses in the nucleus, especially in nuclear genes involved in mitochondrial and plastid interactions (Sloan *et al*., 2014b; Havird *et al*., 2017; Postel *et al*., 2023a). These investigations have found extensive evidence for cytonuclear coevolution and correlated changes in evolutionary rates, which have also been detected in many other angiosperm lineages (Zhang *et al*., 2015; Forsythe *et al*., 2021).

### 3. Mitochondrial gene loss and functional replacement

*Silene* mitogenomes have a low number of protein‐coding and tRNA genes relative to most plants (Sloan *et al*., 2012a), with especially high rates of tRNA gene loss leading to variation among species (Warren *et al*., 2021). Losses of mitochondrial protein‐coding genes in plants are generally mediated by gene transfer to the nucleus (Adams *et al*., 2002). By contrast, tRNA gene losses involve functional replacement via import of cytosolic tRNAs (Warren *et al*., 2021). The extensive variation in mitochondrial tRNA gene content among *Silene* species (e.g. Warren *et al*., 2021) raises questions about how gene replacements affect the network of coevolved interactions between tRNAs and the many enzymes involved in tRNA metabolism. Comparisons among *Silene* species have indicated that mitochondrial tRNA gene loss can be associated with retargeting of enzymes to maintain molecular interactions or with evolution of novel interactions between enzymes and tRNA substrates (Warren *et al*., 2023).

### 4. Heteroplasmy and mode of inheritance for organelle genomes

Although organelle genomes are typically thought to exhibit strict maternal transmission, there is extensive variation in this pattern across plants and other eukaryotes, and some degree of biparental inheritance or ‘paternal leakage’ has been documented for mitogenomes and plastomes in multiple *Silene* species (McCauley, 2013; Postel *et al*., 2024). This mode of inheritance can result in the co‐occurrence of multiple organelle haplotypes in the same individuals (i.e. heteroplasmy), with important implications for other major themes in *Silene* biology, including breeding system evolution and reproductive isolation.

## V. The castrating anther‐smut fungi: plant–pathogen host specialization and coevolution

*Microbotryum* (Basidiomycota, Box 2), a pathogenic fungal complex with more than a hundred species, constitutes, with its host *Silene* species, one of the best characterized natural plant–pathogen systems (Hartmann *et al*., 2019). With more than a hundred species, *Microbotryum* causes the castrating anther‐smut disease. Theoretical studies coupled with long‐term field surveys investigating the ecology of host–pathogen interactions indicated that the anther‐smut disease can limit its host distribution, because the transmission by *Microbotryum* fungi depends both on the frequency and on the density of diseased plants (Uricchio *et al*., 2023). Field studies have also shown that female plants have an advantage against the disease, which can help maintain gynodioecy (Bruns *et al*., 2019).

Most *Microbotryum* species show high host specificity, which raises intriguing questions in terms of adaptation, plant–pathogen coevolution and host specialization (Hartmann *et al*., 2019). Experiments, comparative genomics, population genomics and transcriptomics allowed the study of evolutionary processes across macro‐ and micro‐evolutionary timescales. At the macro‐evolutionary scale, cophylogenies have revealed multiple host‐shifts by the fungi in their evolutionary histories and such shifts have also been regularly observed in the field (Hartmann *et al*., 2019; Box 2). At the micro‐evolutionary scale, within‐species population codivergence is prevalent in many *Silene*‐*Microbotryum* species pairs, likely due to shared historical geographic and climatic constraints, with notably similar glacial refugia (Feurtey *et al*., 2016). *Silene* host plants are more resistant to their endemic pathogens than to foreign pathogens, indicating plant local adaptation (Feurtey *et al*., 2016). Studying *Microbotryum* genomes has revealed that small secreted proteins, often playing a role in pathogenicity, evolved more rapidly than other genes, with footprints of positive selection, yet exhibited little variation in presence/absence within or among species (Beckerson *et al*., 2019). Many selective sweeps have been detected in the anther‐smut genomes, affecting a great part of their genomes, suggesting rapid coevolution with their host plant (Badouin *et al*., 2017). Host proteins that are likely involved in interactions with the fungus have been detected by looking for genes upregulated upon infection and whose protein products interact with fungal proteins (Tsai *et al*., 2024). Experiments showed that resistance to anther‐smut disease varies quantitatively among seed families in *S. vulgaris* (Hood *et al*., 2023), and is controlled by different mechanisms at juvenile and adult stages in *S. latifolia* and *S. vulgaris* (Bruns *et al*., 2022). New methods that jointly analyze host and parasite genomes to identify coevolving loci and reconstruct their coevolutionary history will be a key future research direction in the *Silene*–anther‐smut system (Märkle *et al*., 2021).

## VI. Evolution of sexual systems: from combined to separate sexes

*Silene* is characterized by a high diversity of breeding systems, from cleistogamy to obligate allogamy through dioecy (Casimiro‐Soriguer *et al*., 2015). Therefore, *Silene* constitutes an outstanding model for studying breeding system evolution, in particular, gynodioecy maintenance and mitonuclear control, the transition to dioecy through gynodioecy and selection on sexual dimorphism (e.g. Moquet *et al*., 2022; Barbot *et al*., 2023; Prentout *et al*., 2025). The comparison of the nucleotide diversity between dioecious, gynodioecious and hermaphrodite *Silene* species has revealed that dioecious species exhibit high levels of genetic diversity, with highly effective purifying and positive selection (Muyle *et al*., 2021b). Such higher efficiency of selection in dioecious species may contribute to explain why they do not show higher extinction risks than hermaphrodite species despite expected handicaps such as possible limitations in mates or seed dispersal.

### 1. Gynodioecy as a stable breeding system

There are several conditions for gynodioecy to appear and to be maintained. Females must have a higher fitness than hermaphrodites and sex determination must be under mitonuclear control, in which the effect of mitochondrial factors can be counteracted by nuclear factors (i.e. male fertility restorers). Finally, male fertility restoration must induce a cost; otherwise, restorers will be fixed and sex polymorphism lost (Delph *et al*., 2007). The higher nucleotide diversity of mitochondrial genes observed in gynodioecious compared with nongynodioecious species indicates that balancing selection maintains gynodioecy in *Silene* (Touzet & Delph, 2009). A theoretical model suggests that paternal leakage of mitochondria might favor cytoplasmic diversity and gynodioecy maintenance (Wade & McCauley, 2005). In addition, mitochondrial heteroplasmy might allow the generation of recombinants between mitogenomes and potentially new sterilizing genes (McCauley & Olson, 2008). An open question is whether paternal leakage is more frequent in gynodioecious species or is a constitutive feature in the genus (McCauley, 2013; Postel *et al*., 2024).

Gynodioecious and nongynodioecious species may also differ in the extent of intraspecific mitochondrial genome polymorphism. In the gynodioecious *S. vulgaris*, the mitochondrial genomes are highly rearranged owing to the recombination across numerous large repeats, are fragmented into subgenomes and show no synteny. Only 25% of intergenic sequences are shared among populations, both geographically distant and proximate, all capable of interbreeding (Sloan *et al*., 2012d). By contrast, the dioecious *S. latifolia* possesses a single mitochondrial genome, containing only six major repeats (Sloan *et al*., 2010).

Genetic analyses based on reciprocal crosses and organellar markers indicate the presence of multiple CMS genes and nuclear fertility‐restorer (*Rf*) loci in *S. vulgaris* (Olson *et al*., 2019) and in its sister species *Silene uniflora* (Prentice *et al*., 2011). Although homologous recombination in plant mitochondrial genomes is generally associated with DNA repair, it can also affect the expression of CMS genes and thus male sterility. In the *S. vulgaris* haplotype KRA, recombination has generated two genome configurations. In one configuration, the essential gene *cob* (cytochrome b) is cotranscribed with a CMS factor, whereas in the other configuration, it is expressed independently. Cotranscription constrains the effectiveness of *Rf*‐mediated suppression because inhibiting the CMS gene would simultaneously impair *cob* expression (Štorchová *et al*., 2018). Thus, rearrangements of the mitochondrial genome may influence interactions between nuclear restorers and mitochondrial sterility factors.

Recent advances in mitochondrial genome assembly now allow more detailed structural comparisons across *Silene* species with contrasting breeding systems, which will allow identifying losses and structural rearrangements with functional consequences associated with the evolution of gynodioecy.

### 2. Sexual dimorphism and sexual selection in dioecious species

As observed in many dioecious species (Barrett & Hough, 2013), *Silene* species with separate sexes commonly exhibit sexual dimorphism (i.e. phenotypic differences between males and females). In section *Melandrium* (Fig. 1), sexual dimorphism has been observed across both vegetative and reproductive traits (e.g. timing of flowering, total leaf mass, photosynthetic rate, plant height, corolla size and total flower number) (Delph *et al*., 2010; Barbot *et al*., 2023). Such sexual dimorphism is thought to result from sex‐specific adaptations, which require the resolution of sexual conflicts in order for the independent evolution of male and female traits (Barrett & Hough, 2013). In *S. latifolia*, sexually dimorphic traits typically involve at least one major‐effect quantitative trait locus (QTL) cosegregating with the sex‐determining region, which aligns with the expected outcome of sexually antagonistic selection (Delph *et al*., 2010). In this species, sex‐biased genes make up 16% of the expressed genes in flowers, but only a few percent in leaves (Zemp *et al*., 2016). Most changes in gene expression that led to the observed sex biases in *S. latifolia* happened in the female sex, with most male‐biased genes resulting from a gene expression reduction in females (Zemp *et al*., 2016). A multispecies study on the evolution of sex‐biased gene expression in *Silene* revealed that male‐biased genes appeared first and female‐biased genes second, which might be a consequence of dioecy evolving through the gynodioecy route in this clade, that is, females first and males second. Most changes happened in the female sex, probably because of positive selection, while expression changes in males mainly resulted from drift (Prentout *et al*., 2025).

In insect‐pollinated species, dimorphism may reflect sex‐specific adaptations related to pollinator attraction. Due to anisogamy, male reproductive success is predicted to be more limited by mate acquisition than female reproductive success (Bateman, 1948), as commonly observed in animals (Janicke *et al*., 2016). Efficient pollinator attraction should thus be more advantageous for males than for females, which can maximize their reproductive success with comparatively fewer pollinator visits (Moore & Pannell, 2011). Consistent with theoretical expectations, selection patterns on floral traits in *S. dioica* differed between sexes, both in the intensity of selection and in the identity of traits, in a way that suggests substantial overlap between sexual selection and pollinator‐mediated selection (Barbot *et al*., 2023): In females, traits linked to fecundity (flower and gamete number) had the greatest impact on individual reproductive success, while in males, traits associated with pollinator attraction and mate acquisition (corolla size, flowering duration) were most strongly selected.

Selection patterns and the evolution of sexual dimorphism can be markedly impacted by the ecological context. While sexual dimorphism in attractive floral traits is often considered optimal when pollinators are abundant, it may become disadvantageous when pollinators are scarce, due to disproportionate visitation of the more attractive sex (Vamosi & Otto, 2002) – a prediction experimentally validated in *S. dioica* (Moquet *et al*., 2022). When female reproductive success becomes pollen‐limited, pollinator‐mediated selection on floral traits in females is expected to intensify (Caruso *et al*., 2019), which could lead to a reduction in the degree of sexual dimorphism. Abiotic conditions also influence selection on floral traits. In *S. latifolia*, for instance, males produce more, but smaller flowers than females. Yet, the extent of sexual dimorphism strongly varies across populations (Yu *et al*., 2011), with water availability emerging as a key driver. This is likely because ecophysiological traits are genetically linked to flower size so that selection to reduce water loss favors fewer, larger male flowers in arid environments (Yu *et al*., 2011; Brothers & Delph, 2017).

## VII. Ecological and evolutionary responses to abiotic environments

Research on adaptation to environmental conditions is a fast‐developing field and includes efforts to predict reactions of organisms to ongoing climate change. In evolutionary biology, adaptation is understood as a process by which a population experiences allele frequency shifts that increase its fitness in its habitat (Savolainen *et al*., 2013). Studies in *Silene* have made important and promising contributions, with respect to adaptation to not only extreme conditions (Papadopulos *et al*., 2021) but also mesic conditions (Favre *et al*., 2017; Gramlich *et al*., 2022), and regarding responses to ongoing climate change (Doak & Morris, 2010; DeMarche *et al*., 2018). At the same time, studies on correlations of traits to environmental parameters (Berardi *et al*., 2016; Brothers *et al*., 2016), and phenotypic differentiation and plasticity (del Valle *et al*., 2015; Brothers *et al*., 2016) are indispensable to generate hypotheses regarding adaptive differentiation. With the acquired knowledge on ecological adaptation in native and invasive ranges (e.g. Karrenberg & Favre, 2008) and on genomics (Table 1), *Silene* also represents a good model for studying restoration genetics (Otto, 2018).

### 1. Adaptation to extreme environmental conditions

Tolerance to various heavy metals is common in *Silene*: Both copper and zinc tolerance have evolved after colonization of mine spoils in different *Silene* lineages, for example in *S. vulgaris* (Baloun *et al*., 2014), *Silene paradoxa* (Lazzaro *et al*., 2021) and *S. uniflora* (Papadopulos *et al*., 2021). Recently, population genomic analyses have demonstrated rapid, parallel adaptation to zinc contamination within a single species, *S. uniflora*, with a highly polygenic basis and functional redundancy of the genes recruited in different populations (Papadopulos *et al*., 2021). In addition, plastic responses to salinity in ancestral coastal populations of *S. uniflora* may have facilitated adaptation to zinc contamination in descendant populations (Wood *et al*., 2023; Coates *et al*., 2025). This apparent ‘cross‐talk’ between adaptive responses to different extreme conditions sheds light on an important, but previously underappreciated, evolutionary mechanism that may be more common. Indeed, interacting responses to different stresses in natural habitats, such as high salinity, heavy metals and drought, as well as fungal attack have been demonstrated in *Silene* species (e.g. Papini *et al*., 2019; Wiszniewska *et al*., 2020).

### 2. Long‐term individual‐based monitoring allows for predictions of population shifts

Demographic studies in the cushion plant *S. acaulis*, which occurs in tundra habitats throughout the Northern hemisphere, have led to major advances in the study of adaptation (Doak & Morris, 2010; DeMarche *et al*., 2018). Most species in these habitats are very long‐lived, reaching ages of many decades, if not centuries. For these reasons, population changes develop over long periods of time and are best studied using long‐term individual‐based investigations. Such approaches in plants (Doak & Morris, 2010; Sheldon *et al*., 2022) are now known to provide generally more robust results than comparisons among habitats (space‐for‐time approach; Angert, 2024). In the arctic–alpine *S. acaulis*, populations at the southern range margin had not only reduced seedling recruitment but also increased individual growth, such that a decline will only become apparent once tipping points are reached (Doak & Morris, 2010). Due to local adaptation in *S. acaulis*, global warming may lead to not only range shifts but also a thinning of populations across its range (DeMarche *et al*., 2018); this is a generally understudied aspect of climate change responses. *Silene* contains further species with arctic–alpine distributions and phenological differentiation between populations such as *Silene ciliata* (Morente‐López *et al*., 2020), offering promising study systems for exploration of mechanisms underlying species ranges and ecological responses to changing conditions in tundra environments.

## VIII. Pollinator‐mediated evolution: benefits and costs

*Silene* represents a promising study system in the understanding of pollinator‐mediated floral evolution, in particular flower advertising traits such as floral color, and of the mutualism–parasitism continuum, as *Silene* is one of the few plant systems characterized by nursery pollination (Prieto‐Benítez *et al*., 2017; Berardi *et al*., 2022).

### 1. Pollinator‐mediated floral evolution

Many plant genera are well‐known for the study of plant–pollinator interactions, and especially in the investigation of pollinator‐mediated floral evolution with tight relationships with specific pollinators or pollination modes (e.g. *Mimulus*, *Phlox*, *Petunia*, and *Penstemon*). *Silene*, however, offers a different perspective in that most species can be classified into generalized diurnal, nocturnal, and mixed pollination syndromes. Although *Silene* flowers adhere to (and are possibly constrained by) a similar *bauplan*, there is still considerable floral diversity among species with combinations of floral traits that typically attract hummingbirds, butterflies, bees and flies, nocturnal moths or that promote selfing (Fig. 2; Supporting Information Table S1; Buide *et al*., 2015; Berardi *et al*., 2022). In fact, many *Silene* species are occasionally visited by multiple pollinator guilds (Fig. 2; Table S1). This suggests that floral traits in *Silene* may have evolved to optimize visits from specific pollinator functional groups while still maintaining attractive traits to a diverse range of less efficient pollinators. Retaining features of a generalist pollination syndrome may ensure plant reproductive success through outcrossing in a wide range of environments, for example, when the specialist pollinators are missing or infrequent (Martinell *et al*., 2010; Scopece *et al*., 2018).

Among floral advertising traits, floral color has received considerable attention over the past two decades, positioning *Silene* as a well‐suited system for investigating flower color and pollination. Most *Silene* have either white or pink petals and calyces, with visible color typically based on anthocyanin pigments; infrequent colors in the genus include red and orange (anthocyanins and carotenoids; e.g. *S. regia*, *S. salmonacea*), yellow (carotenoid‐based; e.g. *S. parishii*, *S. otites*) or greenish (carotenoids and chlorophylls; e.g. *S. nutans*) (Figs 1, 2). Red‐flowering *Silene* comprise 1% of the genus and are geographically restricted to regions that overlap with hummingbirds, and have likely shifted their morphology to optimize hummingbird pollination without diverging from the morphological bounds of other North American *Silene* (Berardi *et al*., 2022). Environmental context may also affect floral color evolution. For instance, the anthocyanin pigments responsible for red, pink, purple and blue flower color are not only essential for advertisement of pollinators but also powerful antioxidants and accumulate under plant stress (Dalrymple *et al*., 2020). In Europe, *S. vulgaris* and *Silene littorea* exhibit floral color clines and local adaptation across abiotic gradients such as UV, elevation, precipitation and latitude (del Valle *et al*., 2015; Berardi *et al*., 2016), which might alter pollinator‐mediated selection (Miladin *et al*., 2022).

The multiple studies of phenotypic variance in floral advertising traits (morphology, color, scent and nectar) in experimental and extant populations, combined with pollinator surveys and (phylo)genetic studies, set *Silene* up as a promising genus in which to study more nuanced pollinator‐mediated selection with variable adherence to strict pollination syndromes (Fenster *et al*., 2015). Furthermore, by explicitly linking phenotypic variation to expanded genomic resources (Table 1), future work can also more directly assess the ecological and evolutionary constraints on floral evolution in *Silene*.

### 2. The parasitism–mutualism continuum: nursery pollination

Pollinated plants can constitute an additional reward for pollinating insects by providing breeding sites and food for larvae. In *Silene*, several moth genera, for example *Hadena* (Noctuidae) and *Perizoma* (Geometridae), are nursery (or brood) pollinators, in which females lay eggs in the flowers and caterpillars eat the seeds (Fig. B2 in Box 2; Prieto‐Benítez *et al*., 2017). Moth‐*Silene* host plant specificity is variable, and genetic studies comparing moths and their host plants revealed gene flow asynchrony and complex coevolutionary dynamics, with widespread host shifts (Magalhaes *et al*., 2011; Prieto‐Benítez *et al*., 2017).

Nursery pollination in *Silene* is a remarkable example of an unstable mutualism–parasitism continuum, switching from benefits (increased reproductive success and gene flow) to parasitism costs (seed predation), therefore creating conflicting selective pressures on floral traits (Scopece *et al*., 2018; Zhou *et al*., 2020). Antipredatory strategies to reduce flower attractiveness and access for egg laying and as a response to high fruit predation rate have been highlighted, for example decreased nocturnal scent emission in pollinated flowers, reduced petal size, longer corolla tube, as well as a change in scent and nectar composition and production timing to switch from nocturnal to diurnal pollination (Prieto‐Benítez *et al*., 2017; Martignier *et al*., 2019; Zhou *et al*., 2020). The growing resources of *Silene* genomes (Table 1) offer perspectives for investigating the genetic architecture of those antipredatory strategies and identifying genes involved in parasitism resistance.

## IX. The speciation continuum in *Silene*: understanding how species form

*Silene* is a promising model system for investigating speciation: First, it provides examples of interfertile taxa throughout the speciation continuum, including both closely related and phylogenetically distant taxon pairs (X. Liu *et al*., 2020; Postel *et al*., 2025), and of speciation without obvious ecomorphological differentiation, referred to as cryptic speciation (e.g. Toprak *et al*., 2016). An alternative, potentially instantaneous pathway to speciation is polyploidization, possibly combined with prior interspecific hybridization, with many examples reported in the section *Physolychnis* (Table S2; e.g. Quatela *et al*., 2025). Second, phylogeographic and genetic modeling approaches have inferred whether divergence has occurred with gene flow (sympatry/parapatry; X. Liu *et al*., 2020) or without gene flow (allopatry; Muir *et al*., 2012; Postel *et al*., 2025). Third, a large number of pre‐ and postzygotic barriers have been identified, ranging from flowering time divergence, pollinator‐mediated selection and habitat‐mediated selection to hybrid underperformance and reduced fertility (Table S2). A particular achievement of speciation studies in *Silene* is the use of controlled crosses and manipulative experiments in the natural habitat, in order to identify reproductive barriers acting in natural systems (e.g. Favre *et al*., 2017; Martin *et al*., 2017; Liu & Karrenberg, 2018; Baena‐Díaz *et al*., 2019; Karrenberg *et al*., 2019; Gramlich *et al*., 2022). Finally, studies on *Silene* give important examples of the genetic architecture of traits related to reproductive barriers showing polygenic control, genetic coupling, and an involvement of sex chromosomes (Filatov, 2018; Liu & Karrenberg, 2018; Baena‐Díaz *et al*., 2019). A specifically interesting case is hybrid dysfunction in the heterogametic sex, referred to as Haldane's rule, which was shown in plants for the first time in hybrids between the dioecious *S. diclinis*, *S. latifolia* and *S. dioica* (Brothers & Delph, 2010).

In the last decades, two long‐known *Silene* systems, studied from ecological, phenotypic and genomic perspectives, have made particularly interesting contributions in identifying key evolutionary forces leading to speciation and the genetic control of reproductive barriers between diverging lineages: *S. nutans* and the sister species *S. latifolia* and *S. dioica*.

### 1. Plastid‐nuclear incompatibilities involved in incipient speciation in *Silene nutans*

Recent studies on *S. nutans* have shown that the previously reported morphological and genetic variation across its range (e.g. De Bilde *et al*., 1977) could be translated into several genetically distinct lineages harboring multiple, sometimes asymmetric, pre‐ and postzygotic reproductive isolation barriers (Martin *et al*., 2017; Postel *et al*., 2022, 2025). Those barriers can be associated with allopatry (with genetic lineages having evolved in separate glacial refugia, diverging 760 000–530 000 years ago), temporal isolation in flowering, pollen‐stigma incompatibility, distinct flower scents, F_1_ hybrid seedling chlorosis, low hybrid viability and fertility, and edaphic specialization in one contact zone (Table S2; Postel *et al*., 2022, 2025). The genetic mechanisms behind reproductive isolation are associated with lineage‐specific mutations accumulated in allopatry in fast‐evolving plastid genes and accelerated coevolution of plastid and nuclear genomes (Postel *et al*., 2023a, 2025). The identified functional genes are involved in the gene expression machinery, in particular for plastid and nuclear genes encoding for essential ribosomal proteins and genes in the cytochrome b6/f involved in the photosynthesis process (Postel *et al*., 2022, 2023a). As a consequence, between‐lineage mating leads to plastid‐nuclear incompatibilities, that is, a disruption of plastid‐nuclear protein interactions, resulting in dysfunctional hybrid progeny (Postel *et al*., 2024, 2025). The mitochondrial genome, whose diversity is shaped by gynodioecy and balancing selection, does not contribute to postzygotic reproductive isolation (Postel *et al*., 2023b). The study of the *S. nutans* system for half a century has thus provided an original, elaborated picture of incipient speciation events, delineating morphologically cryptic, but genetically distinct, evolutionary units.

### 2. Speciation with gene flow in *Silene latifolia* and *Silene dioica*

The divergence between white‐flowering *S. latifolia* and pink‐flowering *S. dioica*, which hybridize in nature, is fairly recent, estimated at 0.12 Myr before present, but has led to considerable genetic differentiation with a *F*_ST_ of 0.28 (X. Liu *et al*., 2020). Demographic analyses revealed that divergence with continuous and ongoing gene flow, rather than secondary contact after geographic separation, was the most likely scenario (Muir *et al*., 2012; X. Liu *et al*., 2020). Many incomplete reproductive barriers have been identified, including geographic separation, differential habitat adaptation, flowering time divergence, assortative mating, conspecific pollen precedence, selection against hybrids and fertility reduction in hybrids (Table S2). The two species have largely overlapping Eurasian distributions. *Silene latifolia* is found in drier, warmer and more disturbed habitats than *S. dioica* (Karrenberg & Favre, 2008, and references therein). They share bees and bumblebees as pollinators, whereas the nursery pollinator *Hadena bicruris* is mainly associated with *S. latifolia* (Table S1). The combination of reproductive barriers amounts to near‐complete reproductive isolation, in which ecological barriers, such as habitat adaptation, clearly make the strongest contribution (Karrenberg *et al*., 2019). However, the degree of reproductive isolation is lower under more benign conditions allowing for continued gene flow, as shown in multisite transplant experiments (Karrenberg *et al*., 2019).

These experiments further provided one of the few examples of environmentally dependent hybrid breakdown: second‐generation hybrids performed less well in habitats that exerted stronger selection against the species from the alternative habitat (Favre *et al*., 2017; Karrenberg *et al*., 2019). Fitness proxies in these second‐generation hybrids transplanted into the two habitats were associated with a large number of loci with habitat‐dependent effects that likely constitute barriers to gene flow (Gramlich *et al*., 2022). In general, both vegetative and floral traits associated with reproductive barriers had complex genetic architectures, with QTLs distributed over most chromosomes, including the sex chromosomes (Liu & Karrenberg, 2018; Baena‐Díaz *et al*., 2019). Overall, there is now strong evidence that ecological divergence is the main driver of the speciation process in *S. latifolia* and *S. dioica*.

## X. Conclusions and perspectives

As we have outlined in this review, the *Silene* system offers a unique combination of new and emerging genomic resources, well‐developed experimental expertise, and long‐standing and detailed ecological and phenotypic knowledge in many species. Annotated, chromosome‐level assemblies of nuclear genomes are currently available for five *Silene* species and more are being analyzed (e.g. Cangren *et al*., 2024), while plastomes have been published for 39 species and mitogenomes for four species (Table 1). These resources pave the way for comparative genomic studies as well as for studies on the gene level. For such studies, a fully resolved phylogeny is essential and this is a major goal of current research. Genomic data from species with and without sex chromosomes are also particularly timely and can be used for testing recently proposed theoretical models for the evolution of sex chromosomes, such as neutral processes, heterozygote advantage, differential mutation load and regulatory evolution (Jay *et al*., 2024; Saunders & Muyle, 2024). At the same time, these data can also elucidate the connection between sex chromosome evolution, gene regulation and speciation (Filatov, 2018).

Emerging tools such as CRISPR/Cas9 technology or virus‐induced gene silencing (VIGS) have already been applied in the study of sex determination in *Silene* (Fujita *et al*., 2019; Hudzieczek *et al*., 2019). Such gene editing technologies provide a promising platform for unraveling the genetic underpinnings of cytonuclear interactions, as well as of pathogen resistance or adaptation. Advances in spatial resolution and modern cytogenetic techniques will further enhance studies of genome architecture, bridging the knowledge gap to well‐studied models such as *Arabidopsis thaliana* (Hobza *et al*., 2024). Using these techniques, the functional consequences of the exceptional levels of sequence and structural variation in mitogenomes and plastomes in *Silene*, as described in this review, can be explored. These studies can be extended to focus also on the evolutionary consequences of variation in cytonuclear interactions in which contributions to reproductive isolation between lineages have already been documented (Postel *et al*., 2023b). Interestingly, chloroplast–nuclear interactions are one of the hypotheses for the apparently faster speciation rate in plants as compared to animals (Monnet *et al*., 2025). The *Silene* system offers ideal opportunities for comparative studies to test this and alternative hypotheses, such as divergence in biotic interactions or limited dispersal.

*Silene* harbors many possible cases of repeated evolution in which similar morphologies and putative adaptive differentiation in response to the abiotic habitat or to pollinators arose in distinct groups (Berardi *et al*., 2016; Miladin *et al*., 2022). Analyzing whether genetic variation underlying repeated adaptive evolution stems from new mutations, shared variation or hybridization is a powerful context to gain insight into whether evolution is predictable. Thus far, repeated evolution has been explored within *S. uniflora* (Papadopulos *et al*., 2021). Understanding repeated evolution has strong implications, not only for how biodiversity is generated, but also for predicting possible evolutionary trajectories in the uncertain future of the rapidly changing Anthropocene period and thereby contributing to the preservation and restoration of biodiversity (Otto, 2018). Here, combining studies on adaptation with long‐term field studies, such as those conducted in *S. acaulis* growing in vulnerable arctic habitats (Doak & Morris, 2010), is particularly promising.

We thus foresee that the *Silene* model system will continue to contribute to the investigation of important long‐standing and new questions in evolution and ecology (Box 3), and might be an inspiration for research on other taxonomic groups.

Box 3 **Open questions for which the *Silene* model system is particularly suitable**

1. What are the respective contributions of neutral evolution, regulatory evolution, early dosage compensation, lower mutation load, heterozygote advantage and sexually antagonistic selection to recombination suppression on sex chromosomes?
2. Which genes or gene networks underlie sex determination, cytoplasmic male sterility, host plant resistance to enemies in natural systems, adaptive differentiation and hybrid dysfunction?
3. What are the functional and evolutionary consequences of cytonuclear interactions?
4. What is the source of repeated evolution: standing variation, hybridization or new mutations? Is evolution predictable?
5. What are the expected evolutionary responses of populations to climate change and anthropogenic disturbance?
6. What are the drivers of reproductive isolation?

## Competing interests

None declared.

## Author contributions

SK, VB, AEB, IDC, TG, FEH, RH, VH, GABM, JRM, BO, ASTP, DBS, JCS, HŠ, PT and FVR contributed to the writing and/or critical revisions of the text. SK, PT and FVR led the conceptualization and writing of the manuscript. PT and FVR share last authorship of this work.

## Disclaimer

The New Phytologist Foundation remains neutral with regard to jurisdictional claims in maps and in any institutional affiliations.

## Supporting information


**Notes S1** References cited in Tables 1, S1 and S2.
**Table S1** Pollination syndromes and primary pollinator functional groups in *Silene*, with examples of species. For references, see Notes S1.

**Table S2** Examples of *Silene* systems that have contributed to the understanding of the processes involved in speciation, with genetic divergence, range overlap and hybridization levels, and identified drivers of speciation, pre‐ and postzygotic barriers and related findings. For references, see Notes S1.
Please note: Wiley is not responsible for the content or functionality of any Supporting Information supplied by the authors. Any queries (other than missing material) should be directed to the *New Phytologist* Central Office.
