# Supplementary material for: Silene, a versatile model system: from sex and genome evolution to ecology and speciation
Source: New Phytol. 2026 Apr 5;250(6):3613–30. doi: 10.1111/nph.71153 (PMC13193422; doi:10.1111/nph.71153)
Supplement: Supplementary file 1 — Notes S1 References cited in Tables 1, S1 and S2. Table S1 Pollination syndromes and primary pollinator functional groups in Silene, with examples of species. For references, see Notes S1. [file NPH-250-3613-s002.pdf]

*New Phytologist* **Supporting Information**

**Article title**: *Silene*, a versatile model system: from sex and genome evolution to ecology and speciation

**Authors**: Sophie Karrenberg, Václav Bačovský, Andrea E. Berardi, Isabelle De Cauwer, Tatiana Giraud, Fanny E. Hartmann, Roman Hobza, Vojtěch Hudzieczek, Gabriel A.B. Marais, Jenna R. Miladin, Bengt Oxelman, Alexander S.T. Papadopulos, Daniel B. Sloan, Janet C. Steven, Helena Štorchová, Pascal Touzet and Fabienne Van Rossum

**Article acceptance date:** 13 March 2026.

The following Supporting Information is available for this article:

**Table S1** Pollination syndromes and primary pollinator functional groups in *Silene.*

**Table S2** Speciation studies in *Silene* (see separate Excel file).

**Note S1** References cited in Table 1 and in Supplementary Tables S1 and S2.

**Table S1** Pollination syndromes and primary pollinator functional groups in *Silene*, with examples of species. For references, see Note S1.

| **Pollination syndrome** | **Main pollinator groups** | **Examples of *Silene* species** | **References (not exhaustive)** |
|---|---|---|---|
| Diurnal pollination | Hummingbirds and hawkmoths | *S. virginica* | Reynolds *et al.*, 2009; Berardi *et al.*, 2022 |
| Diurnal pollination | Bees, flies and/or butterflies | *S. caroliniana, S. diclinis, S. spaldingii* | Reynolds *et al.*, 2009; Brothers & Atwell, 2014; Berardi *et al.*, 2022; Hatten *et al.*, 2024 |
| Nocturnal pollination | Moths including nursery pollinators | *S. latifolia, S. niceensis, S. nutans, S. sennenii, S. stellata* | Reynolds *et al.*, 2009, 2012; Labouche & Bernasconi, 2010; Martinell *et al.*, 2010; Brothers & Atwell, 2014; Magalhaes & Bernasconi, 2014; Buide *et al.*, 2015; Prieto-Benítez *et al.*, 2017; Zhou *et al.*, 2018; Vanderplanck *et al.*, 2020; Cornet *et al.*, 2022; Berardi *et al.*, 2022 |
| Mixed pollination system | Diurnal-nocturnal including nursery pollinators | *S. dioica, S. latifolia, S. otites, S. vulgaris, S. colorata, S. noctiflora* | Goulson & Jerrim, 1997; Waelti *et al.*, 2008; Dötterl *et al.*, 2012; Witt *et al.*, 2013; Page *et al.*, 2014; Scopece *et al.*, 2018; Cortés-Giménez *et al.*, 2024; Barbot *et al.*, 2025 |
| Selfing | | *S. antirrhina, S. ramosissima, S. apetala, S. nocturna, S. gallica, S. inaperta, S, uralensis, S. sedoides* | Buide *et al.*, 2015; Berardi *et al.*, 2022; Quatela *et al.*, 2025 |

**Table S2** See separate Excel file.

**Note S1** References cited in the Supplementary Tables.

**Andersson S, Månsby E, Prentice HC**. **2008**. Paternal effects on seed germination: a barrier to the genetic assimilation of an endemic plant taxon? *Journal of Evolutionary Biology* **21**: 1408–1417.

**Barbot E, Dufaÿ M, Godé C, De Cauwer I**. **2025**. Investigating the effects of diurnal and nocturnal pollinators male female reproductive success and on floral trait selection in *Silene dioica*. *Peer Community Journal* **5**: e11.

**Berardi AE, Betancourt Morejón AC, Hopkins R**. **2022**. Convergence without divergence in North American red-flowering *Silene*. *Frontiers in Plant Science* **13**: 945806.

**Bratteler M, Lexer C, Widmer A**. **2006**. Genetic architecture of traits associated with serpentine adaptation of *Silene vulgaris*. *Journal of Evolutionary Biology* **19**: 1149–1156.

**Brothers AN, Atwell JW**. **2014**. The role of pollinator-mediated selection in the divergence of floral traits between two closely related plant species. *International Journal of Plant Sciences* **175**: 287–295.

**Brothers AN, Delph LF**. **2010**. Haldane's rule is extended to plants with sex chromosomes. *Evolution* **64**: 3643–3648.

**Bruun HH, Van Rossum F, Ström L**. **2001**. Exudation of low molecular weight organic acids by germinating seeds of two edaphic ecotypes of *Silene nutans* L. *Acta Oecologica* **22**: 285–291.

**Buide ML, del Valle JC, Pissatto M, Narbona E**. **2015**. Night life on the beach: selfing to avoid pollinator competition between two sympatric *Silene* species. *Annals of Botany* **116**: 201–211.

**Cornet C, Noret N, Van Rossum F**. **2022**. Pollinator sharing between reproductively isolated genetic lineages of *Silene nutans*. *Frontiers in Plant Science* **13**: 927498.

**Cortés-Giménez FD, Far AJ, Cerrato MD, Pinya S, Cursach J**. **2024**. First results about pollination of *Silene migjornensis*, an endemic plant species of the southern dunes of Mallorca. *Journal of Applied Entomology* **149**: 882–887.

**Davy AJ, Baker AJM, Willis AJ**. **2024**. Biological Flora of Britain and Ireland: *Silene uniflora*. *Journal of Ecology* **112**: 2135–2157.

**De Bilde J**. **1973**. Etude génécologique du *Silene nutans* L. en Belgique: populations du *Silene nutans* L. sur substrats siliceux et calcaires. *Revue Générale de Botanique* **80**: 161–176.

**De Bilde J**. **1977**. Effet de l'aluminium et du calcium sur l'activité phosphatasique acide des racines d'écotypes calcicole et silicole de *Silene nutans* L. (Caryophyllacées). *Bulletin de la Société Royale de Botanique de Belgique* **110**: 151–160.

**De Bilde J**. **1978**. Nutrient adaptation in native and experimental calcicolous and siliceous populations of *Silene nutans*. *Oikos* **31**: 383.

**De Bilde J, Briane JP, Gorenflot R**. **1977**. Traitement numérique de populations du *Silene nutans* L. d'Europe nord-occidentale et centrale. *Revue Générale de Botanique* **84**: 341–354.

**De Bilde J, Lefèbvre C**. **1990**. The strategy of *Silene nutans* on calcareous and siliceous soils. *Acta Oecologica* **11**: 399–408.

**Demuth JP, Flanagan RJ, Delph LF**. **2014**. Genetic architecture of isolation between two species of *Silene* with sex chromosomes and Haldane's rule: Genetic architecture of reproductive isolation. *Evolution* **68**: 332–342.

**Dötterl S, Jahreiß K, Jhumur US, Jürgens A**. **2012**. Temporal variation of flower scent in *Silene otites* (Caryophyllaceae): a species with a mixed pollination system. *Botanical Journal of the Linnean Society* **169**: 447–460.

**Favre A, Widmer A, Karrenberg S**. **2017**. Differential adaptation drives ecological speciation in campions (*Silene*): evidence from a multi-site transplant experiment. *The New Phytologist* **213**: 1487–1499.

**Goulson D**. **2009**. Evaluating the role of ecological isolation in maintaining the species boundary between *Silene dioica* and *S. latifolia*. *Plant Ecology* **205**: 201–211.

**Goulson D, Jerrim K**. **1997**. Maintenance of the species boundary between *Silene dioica* and *S. latifolia* (red and white campion). *Oikos* **79**: 115.

**Gramlich S, Liu X, Favre A, Buerkle CA, Karrenberg S**. **2022**. A polygenic architecture with habitat-dependent effects underlies ecological differentiation in *Silene*. *The New Phytologist* **235**: 1641–1652.

**Hatten TD, Griswold T, Gibbs J**. **2024**. Spatiotemporal variability and foraging behavior of bee visitors to a rare long-lived iteroparous forb, *Silene spaldingii* (Caryophyllaceae). *Scientific Reports* **14**: 24667.

**Howell EC, Armstrong SJ, Filatov DA**. **2009**. Evolution of neo-sex chromosomes in *Silene diclinis*. *Genetics* **182**: 1109–1115.

**Hu X-S, Filatov DA**. **2016**. The large-X effect in plants: increased species divergence and reduced gene flow on the *Silene* X-chromosome. *Molecular Ecology* **25**: 2609–2619.

**Ironside JE, Filatov DA**. **2005**. Extreme population structure and high interspecific divergence of the *Silene* Y chromosome. *Genetics* **171**: 705–713.

**Karrenberg S, Favre A**. **2008**. Genetic and ecological differentiation in the hybridizing campions *Silene dioica* and *S. latifolia*. *Evolution* **62**: 763–773.

**Karrenberg S, Liu X, Hallander E, Favre A, Herforth-Rahmé J, Widmer A**. **2019**. Ecological divergence plays an important role in strong but complex reproductive isolation in campions (*Silene*). *Evolution* **73**: 245–261.

**Labouche A-M, Bernasconi G**. **2010**. Male moths provide pollination benefits in the *Silene latifolia–Hadena bicruris* nursery pollination system: sex-specific pollination in a dioecious plant. *Functional Ecology* **24**: 534–544.

**Liu X, Glémin S, Karrenberg S**. **2020**. Evolution of putative barrier loci at an intermediate stage of speciation with gene flow in campions (*Silene*). *Molecular Ecology* **29**: 3511–3525.

**Liu X, Karrenberg S**. **2018**. Genetic architecture of traits associated with reproductive barriers in *Silene*: Coupling, sex chromosomes and variation. *Molecular Ecology* **27**: 3889–3904.

**Magalhaes IS, Bernasconi G**. **2014**. Phenotypic divergence and inter-specific trait correlation in a plant-pollinator/seed predator mutualism. *Evolutionary Ecology* **28**: 905–922.

**Martin H, Touzet P, Dufay M, Godé C, Schmitt E, Lahiani E, Delph LF, Van Rossum F**. **2017**. Lineages of *Silene nutans* developed rapid, strong, asymmetric postzygotic reproductive isolation in allopatry. *Evolution* **71**: 1519–1531.

**Martin H, Touzet P, Van Rossum F, Delalande D, Arnaud J-F**. **2016**. Phylogeographic pattern of range expansion provides evidence for cryptic species lineages in *Silene nutans* in Western Europe. *Heredity* **116**: 286–294.

**Martinell MC, Dötterl S, Blanché C, Rovira A, Massó S, Bosch M**. **2010**. Nocturnal pollination of the endemic *Silene sennenii* (Caryophyllaceae): an endangered mutualism? *Plant Ecology* **211**: 203–218.

**Montgomery BR, Soper DM, Delph LF**. **2010**. Asymmetrical conspecific seed-siring advantage between *Silene latifolia* and *S. dioica*. *Annals of Botany* **105**: 595–605.

**Moyle LC, Olson MS, Tiffin P**. **2004**. Patterns of reproductive isolation in three angiosperm genera. *Evolution* **58**: 1195.

**Muir G, Dixon CJ, Harper AL, Filatov DA**. **2012**. Dynamics of drift, gene flow, and selection during speciation in *Silene*. *Evolution* **66**: 1447–1458.

**Nista P, Brothers AN, Delph LF**. **2015**. Differences in style length confer prezygotic isolation between two dioecious species of *Silene* in sympatry. *Ecology and Evolution* **5**: 2703–2711.

**Page P, Favre A, Schiestl FP, Karrenberg S**. **2014**. Do flower color and floral scent of *Silene* species affect host preference of *Hadena bicruris*, a seed-eating pollinator, under field conditions? *PloS One* **9**: e98755.

**Petri A, Oxelman B. 2011.** Phylogenetic relationships within *Silene* (Caryophyllaceae) section *Physolychnis*. *Taxon* **60**: 953–968.

**Pfeil BE, Toprak Z, Oxelman B**. **2017**. Recombination provides evidence for ancient hybridisation in the *Silene aegyptiaca* (Caryophyllaceae) complex. *Organisms, Diversity & Evolution* **17**: 717–726.

**Popp M, Erixon P, Eggens F, Oxelman B**. **2005**. Origin and evolution of a circumpolar polyploid species complex in *Silene* (Caryophyllaceae) inferred from low copy nuclear RNA polymerase introns, rDNA, and chloroplast DNA. *Systematic Botany* **30**: 302–313.

**Popp M, Oxelman B**. **2007**. Origin and evolution of North American polyploid *Silene* (Caryophyllaceae). *American Journal of Botany* **94**: 330–349.

**Postel Z, Martin H, Roux C, Godé C, Genete M, Schmitt É, Monnet F, Vekemans X, Touzet P**. **2025**. Rapid genetic isolation among four lineages of *Silene nutans*. *Plant and Cell Physiology* **66**: 514–528.

**Postel Z, Mauri T, Lensink MF, Touzet P**. **2023a**. What is the potential impact of genetic divergence of plastid ribosomal genes between *Silene nutans* lineages in hybrids? An in silico approach using the 3D structure of the plastid ribosome. *Frontiers in Plant Science* **14**: 1167478.

**Postel Z, Poux C, Gallina S, Varré J-S, Godé C, Schmitt E, Meyer E, Van Rossum F, Touzet P**. **2022**. Reproductive isolation among lineages of *Silene nutans* (Caryophyllaceae): A potential involvement of plastid-nuclear incompatibilities. *Molecular Phylogenetics and Evolution* **169**: 107436.

**Postel Z, Sloan DB, Gallina S, Godé C, Schmitt E, Mangenot S, Drouard L, Varré J-S, Touzet P**. **2023b**. The decoupled evolution of the organellar genomes of *Silene nutans* leads to distinct roles in the speciation process. *The New Phytologist* **239**: 766–777.

**Postel Z, Van Rossum F, Godé C, Schmitt É, Touzet P**. **2024**. Paternal leakage of plastids rescues inter-lineage hybrids in *Silene nutans*. *Annals of Botany* **133**: 427–434.

**Prentice HC, Andersson S, Månsby E**. **2011**. Mosaic variation in allozyme and plastid DNA markers in the European ranges of *Silene vulgaris* and its partially sympatric relative *S. uniflora* (Caryophyllaceae). *Botanical Journal of the Linnean Society* **166**: 127–148.

**Prieto-Benítez S, Yela JL, Giménez-Benavides L**. **2017**. Ten years of progress in the study of *Hadena*-Caryophyllaceae nursery pollination. A review in light of new Mediterranean data. *Flora* **232**: 63–72.

**Quatela A-S, Cangren P, de Lima Ferreira P, Woudstra Y, Zsoldos-Skahjem A, Bacon CD, de Boer HJ, Oxelman B**. **2025**. Phylogenetic relationships and the identification of allopolyploidy in circumpolar *Silene* sect. *Physolychnis*. *American Journal of Botany* **112**: e70051.

**Rahmé J, Widmer A, Karrenberg S**. **2009**. Pollen competition as an asymmetric reproductive barrier between two closely related *Silene* species. *Journal of Evolutionary Biology* **22**: 1937–1943.

**Reynolds RJ, Kula AAR, Fenster CB, Dudash MR**. **2012**. Variable nursery pollinator importance and its effect on plant reproductive success. *Oecologia* **168**: 439–448.

**Reynolds RJ, Westbrook MJ, Rohde AS, Cridland JM, Fenster CB, Dudash MR**. **2009**. Pollinator specialization and pollination syndromes of three related North American *Silene*. *Ecology* **90**: 2077–2087.

**Runyeon-Lager H, Prentice HC**. **2000**. Morphometric variation in a hybrid zone between the weed, *Silene vulgaris,* and the endemic, *Silene uniflora* ssp. *petraea* (Caryophyllaceae), on the Baltic island of Öland. *Canadian Journal of Botany* **78**: 1384–1397.

**Runyeon H, Prentice HC**. **1997**. Patterns seed polymorphism and allozyme variation in the bladder campions, *Silene vulgaris* and *Silene uniflora* (Caryophyllaceae). *Canadian Journal of Botany* **75**: 1868–1886.

**Scopece G, Campese L, Duffy KJ, Cozzolino S**. **2018**. The relative contribution of diurnal and nocturnal pollinators to plant female fitness in a specialized nursery pollination system. *AoB Plants* **10**: ly002.

**Spadafora ND, Eggermont D, Křešťáková V, Chenet T, Van Rossum F, Purcaro G**. **2023**. Comprehensive analysis of floral scent and fatty acids in nectar of *Silene nutans* through modern analytical gas chromatography techniques. *Journal of Chromatography A* **1696**: 463977.

**Toprak Z, Pfeil BE, Jones G, Marcussen T, Ertekin AS, Oxelman B**. **2016**. Species delimitation without prior knowledge: DISSECT reveals extensive cryptic speciation in the *Silene aegyptiaca* complex (Caryophyllaceae). *Molecular Phylogenetics and Evolution* **102**: 1–8.

**del Valle JC, Casimiro-Soriguer I, Buide ML, Narbona E, Whittall JB**. **2019**. Whole plastome sequencing within *Silene* section *Psammophilae* reveals mainland hybridization and divergence with the Balearic Island populations. *Frontiers in Plant Science* **10**.

**Vanderplanck M, Touzet P, Van Rossum F, Lahiani E, De Cauwer I, Dufaÿ M**. **2020**. Does pollination syndrome reflect pollinator efficiency in *Silene nutans*? *Acta Oecologica* **105**: 103557.

**Van Rossum F, De Bilde J, Lefèbvre C**. **1996**. Barriers hybridization calcicolous silicicolous populations *Silene nutans* from Belgium. *Belgian Journal of Botany* **129**: 13–18.

**Van Rossum F, Martin H, Le Cadre S, Brachi B, Christenhusz MJM, Touzet P**. **2018**. Phylogeography of a widely distributed species reveals a cryptic assemblage of distinct genetic lineages needing separate conservation strategies. *Perspectives in Plant Ecology, Evolution and Systematics* **35**: 44–51.

**Van Rossum F, Meerts P, Gratia E, Tanghe M. 1999**. Ecological amplitude in *Silene nutans* in relation to allozyme variation at the western margin of its distribution. *Journal of Vegetation Science* **10**: 253–260.

**Waelti MO, Muhlemann JK, Widmer A, Schiestl FP. 2008**. Floral odour and reproductive isolation in two species of *Silene*. *Journal of Evolutionary Biology* **21**: 111–121.

**Weingartner LA, Delph LF. 2014**. Neo-sex chromosome inheritance across species in *Silene* hybrids. *Journal of Evolutionary Biology* **27**: 1491–1499.

**Witt T, Jürgens A, Gottsberger G. 2013**. Nectar sugar composition of European Caryophylloideae (Caryophyllaceae) in relation to flower length, pollination biology and phylogeny. *Journal of Evolutionary Biology* **26**: 2244–2259.

**Zhang J-J, Montgomery BR, Huang S-Q. 2016**. Evidence for asymmetrical hybridization despite pre- and post-pollination reproductive barriers between two *Silene* species. *AoB Plants* **8**: lw032.

**Zhou J, Zimmer EA, Fenster CB, Dudash MR. 2018**. Characterization of the mating system of a native perennial tetraploid herb, *Silene stellata*. *American Journal of Botany* **105**: 1643–1652.
